# Supplementary material for: DNA strand breaks and gaps target retroviral intasome binding and integration
Source: Nat Commun. 2023 Nov 3;14:7072. doi: 10.1038/s41467-023-42641-4 (PMC10624929; doi:10.1038/s41467-023-42641-4)
Supplement: Supplementary file 1 — Supplementary Information [file 41467_2023_42641_MOESM1_ESM.pdf]

Supplementary Information

DNA Strand Breaks and Gaps Target Retroviral Intasome Binding and Integration

Gayan Senavirathne[1], James London[1], Anne Gardner[1], Richard Fishel[1,2,*] and Kristine E. Yoder[1,2,*]

[1]Cancer Biology and Genetics, The Ohio State University College of Medicine, Columbus, OH 43210

[2]Molecular Carcinogenesis and Chemoprevention Program, The James Comprehensive Cancer Center and Ohio State University, Columbus, OH 43210, US

*Correspondence may also be addressed to: R. Fishel (fishel.7@osu.edu) or K.E. Yoder (yoder.176@osu.edu)

Supplementary Table 1. Target DNAs and vDNAs Used in this Study.

Oligo names	Sequence	DNA Substrate
KEY22<br>KEY21	5′-Bio-CTGGAGAATCCCGGTGCCGAGGCCGCTCAATTGGTCGTAGACAGCTCTAGCACCGCTTAA-3′<br>3′-GACCTCTTAGGGCCACGGCTCCGGCGAGTTAACCAGCATCTGTCGAGATCGTGGCGAATT-5′	GC
KEY25<br>KEY21	8-OxoG<br>5′-Bio-CTGGAGAATCCCGGTGCCGAGGCCGCTCAATTGGTCGTAGACAGCTCTAGCACCGCTTAA-3′<br>3′-GACCTCTTAGGGCCACGGCTCCGGCGAGTTAACCAGCATCTGTCGAGATCGTGGCGAATT-5′	8-oxo-G
KEY22<br>KEY28	5′-Bio-CTGGAGAATCCCGGTGCCGAGGCCGCTCAATTGGTCGTAGACAGCTCTAGCACCGCTTAA-3′<br>3′-GACCTCTTAGGGCCACGGCTCCGGCGAGTTAACCAGCATTTGTCGAGATCGTGGCGAATT-5′	G/T
KEY29<br>KEY21	T<br>5′-Bio-CTGGAGAATCCCGGTGCCGAGGCCGCTCAATTGGTCGTAGACAGCTCTAGCACCGCTTAA-3′<br>3′-GACCTCTTAGGGCCACGGCTCCGGCGAGTTAACCAGCATCTGTCGAGATCGTGGCGAATT-5′	+T
KEY23 KEY24(5'-P)<br>KEY 21	HO P<br>5′-Bio-CTGGAGAATCCCGGTGCCGAGGCCGCTCAATTGGTCGTAGACAGCTCTAGCACCGCTTAA-3′<br>3′-GACCTCTTAGGGCCACGGCTCCGGCGAGTTAACCAGCATCTGTCGAGATCGTGGCGAATT-5′	Nick (5'-P)
KEY23 KEY26(5'-P)<br>KEY 21	HO P<br>5′-Bio-CTGGAGAATCCCGGTGCCGAGGCCGCTCAATTGGTCGTA ACAGCTCTAGCACCGCTTAA-3′<br>3′-GACCTCTTAGGGCCACGGCTCCGGCGAGTTAACCAGCATCTGTCGAGATCGTGGCGAATT-5′	1nt Gap (5'-P)
KEY27 KEY26(5'-P)<br>KEY 21	HO P<br>5′-Bio-CTGGAGAATCCCGGTGCCGAGGCCGCTCAATTGGTCGT ACAGCTCTAGCACCGCTTAA-3′<br>3′-GACCTCTTAGGGCCACGGCTCCGGCGAGTTAACCAGCATCTGTCGAGATCGTGGCGAATT-5′	2nt Gap (5'-P)
KEY23 KEY24(5'-OH)<br>KEY 21	HO OH<br>5′-Bio-CTGGAGAATCCCGGTGCCGAGGCCGCTCAATTGGTCGTAGACAGCTCTAGCACCGCTTAA-3′<br>3′-GACCTCTTAGGGCCACGGCTCCGGCGAGTTAACCAGCATCTGTCGAGATCGTGGCGAATT-5′	Nick (5'-OH)
KEY23 KEY26(5'-OH)<br>KEY 21	HO OH<br>5′-Bio-CTGGAGAATCCCGGTGCCGAGGCCGCTCAATTGGTCGTA ACAGCTCTAGCACCGCTTAA-3′<br>3′-GACCTCTTAGGGCCACGGCTCCGGCGAGTTAACCAGCATCTGTCGAGATCGTGGCGAATT-5′	1nt Gap (5'-OH)
KEY27 KEY26(5'-OH)<br>KEY 21	HO OH<br>5′-Bio-CTGGAGAATCCCGGTGCCGAGGCCGCTCAATTGGTCGT ACAGCTCTAGCACCGCTTAA-3′<br>3′-GACCTCTTAGGGCCACGGCTCCGGCGAGTTAACCAGCATCTGTCGAGATCGTGGCGAATT-5′	2nt Gap (5'-OH)
KEY23 Cy5-KEY26(5'-OH)<br>KEY 21	HO OH<br>5′-Bio-CTGGAGAATCCCGGTGCCGAGGCCGCTCAATTGGTCGTA ACAGCTCTAGCACCGCTTAA-3′<br>3′-GACCTCTTAGGGCCACGGCTCCGGCGAGTTAACCAGCATCTGTCGAGATCGTGGCGAATT-5′	R-Cy5<br>1nt Gap (5'-OH)
KEY675<br>Cy3-KEY616	5′-CTGTTCGGGCGCCACTCAATATACAAAATTCCATGACA-3′<br>3′-GACAAGCCCGCGGTGAGTTATATGTTTTAAGGTACTGTTA-5′	Cy3-PFV
3'-ddA KEY675<br>Cy3-KEY616	5′-CTGTTCGGGCGCCACTCAATATACAAAATTCCATGAC[2′3′ddA]-3′<br>3′-GACAAGCCCGCGGTGAGTTATATGTTTTAAGGTACTGTTA-5′	Cy3-PFV-ddA
KEY675<br>KEY616	5′-CTGTTCGGGCGCCACTCAATATACAAAATTCCATGACA-3′<br>3′-GACAAGCCCGCGGTGAGTTATATGTTTTAAGGTACTGTTA-5′	PFV
Cy3-KEY675(2)<br>KEY616	5′-GCGCCACTCAATATACAAAATTCCATGACA-3′<br>3′-CGCGGTGAGTTATATGTTTTAAGGTACTGTTA-5′	Cy3-PFV (TS)
KEY675<br>Cy5-KEY616	5′-CTGTTCGGGCGCCACTCAATATACAAAATTCCATGACA-3′<br>3′-GACAAGCCCGCGGTGAGTTATATGTTTTAAGGTACTGTTA-5′	Cy5-PFV

The constituent DNA oligonucleotides, the target DNA substrates, and the vDNAs used in this study. The labeling positions of AF488, Cy3 and Cy5 are marked with blue, green, and red colors respectively. Bio = 5'-Biotin, OH = Hydroxyl, P = Phosphate. Target DNA containing a Cy5 fluorophore located on the undamaged strand, 10 or 11 bp to the 5'-side relative to the lesion site is labeled **F-Cy5** in **Fig. 4d** and **Supplementary Fig. 11**.

Supplementary Table 2. Statistics for Pseudo-FRET from the Target Capture Complex Histograms Recorded at 100 ms Frame Rate.

DNA Substrate	Number of DNA molecules (N)	Pseudo FRET	Pseudo FRET
		Total number of counts (n)	Number of counts per DNA (n/N)
GC	552	283	0.513
8-oxo-G	496	258	0.521
G/T	574	342	0.596
+T	606	442	0.730
Nick (5'-P)	549	314	0.571
1nt Gap (5'-P)	540	381	0.706
2nt Gap (5'-P)	474	322	0.680
Nick (5'-OH)	558	346	0.621
1nt Gap (5'-OH)	496	356	0.718
2nt Gap (5'-OH)	540	194	0.359
Blocked end 1nt Gap (5'-OH)	483	297	0.614

$\bar{\boldsymbol{x}} = 0.603, \sigma = \pm 0.110$

These numbers were calculated as described in the **METHODS** by fitting FRET distributions for each DNA with a single or a combination of two Gaussians and integrating the area under the curve for the 0.06 FRET peak. $\bar{\boldsymbol{x}}$ and σ show the average and the standard deviation for the counts per DNA calculated from all the DNA substrates.

Supplementary Table 3. Statistics for Pseudo-FRET from the Target Capture Complex Histograms Recorded at 1 s Frame Rate.

DNA Substrate	Number of DNA molecules (N)	Pseudo FRET	Pseudo FRET
		Total number of counts (n)	Number of counts per DNA (n/N)
GC	583	606	1.04
8-oxo-G	624	2359	3.78
G/T	613	1765	2.88
+T	531	1529	2.88
Nick (5'-P)	583	2268	3.89
1nt Gap (5'-P)	542	2000	3.69
2nt Gap (5'-P)	498	1604	3.22
Nick (5'-OH)	614	1105	1.8
1nt Gap (5'-OH)	489	2308	4.72
2nt Gap (5'-OH)	534	951	1.78

$\bar{\boldsymbol{x}} = 2.97, \sigma = \pm 1.14$

These numbers were calculated as described in the **METHODS** by fitting FRET distributions for each DNA with a single or a combination of two Gaussians and integrating the area under the curve for the 0.06 FRET peak. $\bar{x}$ and σ show the average and the standard deviation for the counts per DNA calculated from all the DNA substrates.

Supplementary Table 4. Strand Transfer Times.

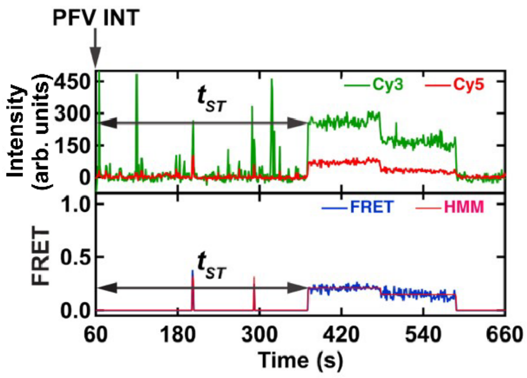

Cy3-PFV

DNA Substrate	$\bar{t}_{ST} \pm \sigma_{ST}$ (s)	Number of events (n)
GC	172 ± 87	2
G/T	328 ± 228	3
Nick (5'-P)	191 ± 143	4
1nt Gap (5'-P)	254 ± 202	17
2nt Gap (5'-P)	264 ± 176	7
Nick (5'-OH)	180 ± 147	12
1nt Gap (5'-OH)	249 ± 150	125
2nt Gap (5'-OH)	277 ± 148	71
Blocked end 1nt Gap (5'-OH)	260 ± 145	134
R-Cy5 1nt Gap (5'-OH)	216 ± 152	104

Cy3-PFV (TS1)

DNA Substrate	$\bar{t}_{ST} \pm \sigma_{ST}$ (s)	Number of events (n)
1nt Gap (5'-P)	229 ± 166	28
1nt Gap (5'-OH)	255 ± 159	124

Cy3-PFV-ddA

DNA Substrate	$\bar{t}_{ST} \pm \sigma_{ST}$ (s)	Number of events (n)
1nt Gap (5'-OH)	186 ± 103	2

Cy3/Cy5-PFV

DNA Substrate	$\bar{t}_{ST} \pm \sigma_{ST}$ (s)	Number of events (n)
GC	140 ± 38	2
1nt Gap (5'-OH)	251 ± 156	95

The strand transfer time (t_{ST}) is the time it takes to observe a stable FRET (or Cy3) signal from the intasome injection, as shown by the figure in the first raw. The mean values ($\bar{t}$) and standard deviations (σ) were calculated as described in the **Methods**. *n* indicates the number of DNAs that showed strand transfer events. Source data are provided in the Source Data File.

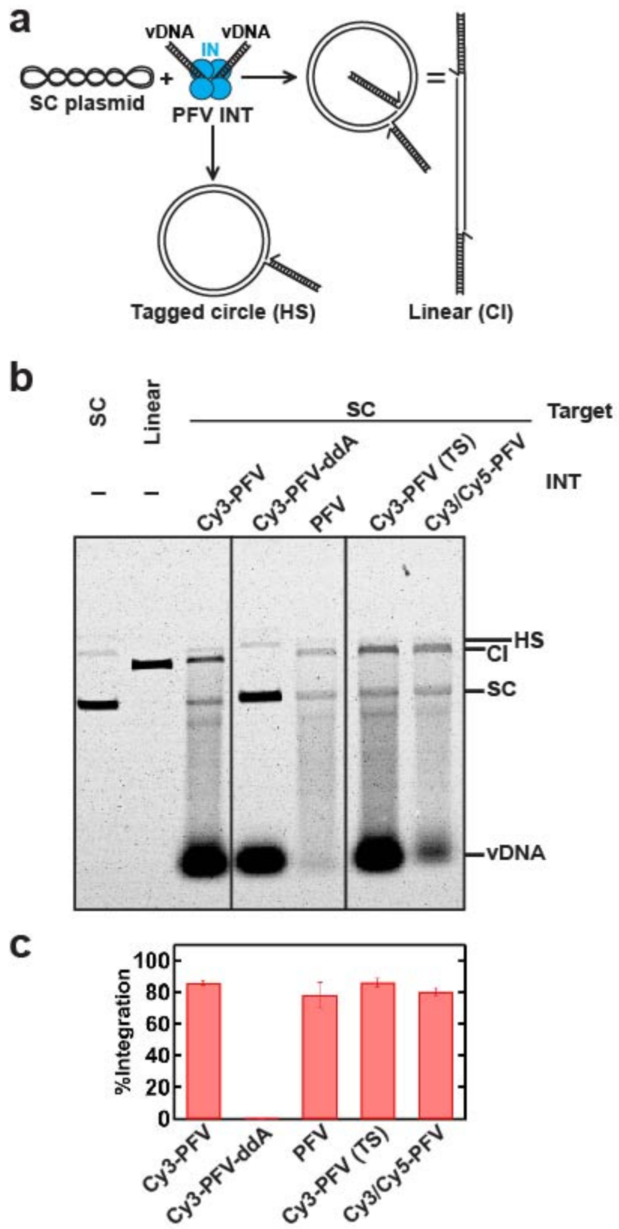

Supplementary Figure 1. Integration Activity of PFV Intasome Preparations. (**a**) An illustration of the gel-based integration analysis. When a PFV intasome (INT) consisting of PFV integrase (IN) and oligonucleotides mimicking viral DNA (vDNA) ends catalyzes concerted integration (CI) into a supercoiled (SC) DNA, a linear DNA is produced. A relaxed tagged circle is created when one strand transfer occurs in a half-site (HS) integration. (**b**) An ethidium bromide-stained agarose gel showing the integration activity of all the intasomes used in this study. Integration initially results in a linear CI product. Secondary integration events result in a smear of smaller products that extend from the linear CI to the vDNA. Competing intasome inactivation and/or aggregation deplete the active pool and cause the incomplete conversion of the SC DNA to CI products. (**c**) Quantification of integration activity. Error bars indicate standard deviation from two independent experiments. HS products that migrate slower than the linear DNA were undetectable with ethidium bromide. The Cy3-PFV-ddA did not produce any detectable activity. Secondary integration events that resulted in smearing below the linear CI products, excluding the SC band, were quantified and added to the linear CI product for this analysis. Source data are provided in the Source Data File.

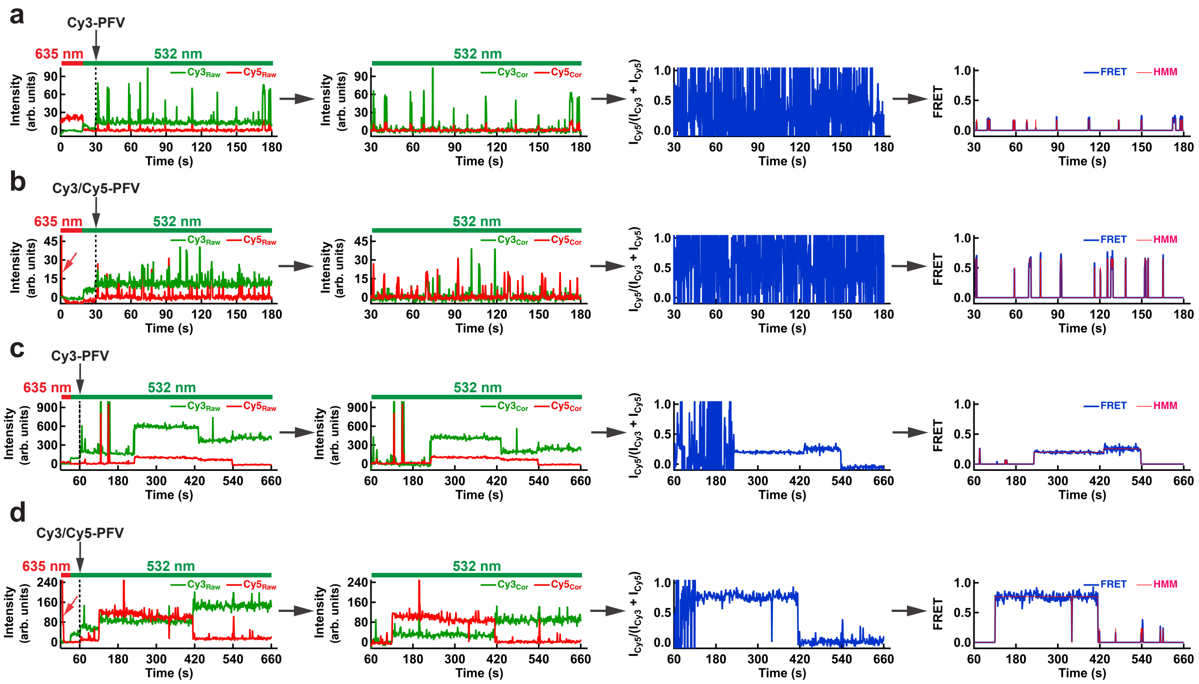

Supplementary Figure 2. The Algorithm Used to Truncate, Background Correct, and Perform HMM Analysis of the smFRET Trajectories. (**a**) A representative TCC trajectory for Cy3-PFV intasomes interacting with a fully duplex DNA collected at 100 ms frame rate. The raw intensity trajectory shows the initial 635 nm laser excitation, switching to the 532 nm laser, and real-time intasome injection (Panel 1). The beginning part of the raw intensity trajectory was truncated at the intasome injection, and Cy3, Cy5 emission backgrounds were corrected (Panel 2). The ratiometric FRET for the truncated trajectory shows erratic fluctuations when both Cy3 and Cy5 signals approach zero (Panel 3). Therefore, zero values were assigned for calculated FRET and the HMM fitting when Cy3 or Cy5 intensity reaches the background noise level (Panel 4). (**b**) A representative TCC trajectory for Cy3/Cy5-PFV intasomes interacting with a 1 nt Gap (5'-OH) collected at 100 ms frame rate. Similar analysis was performed as described in (**a**), except the initial 635 laser exposure was used to photobleach the Cy5 emission on the substrate (red arrow in Panel 1). (**c**) A representative STC trajectory for Cy3-PFV intasomes with a 1 nt Gap (5'-OH) collected at 1 s frame rate. Similar analysis was performed as described in (**a**). (**d**) A representative STC trajectory for Cy3/Cy5-PFV intasomes with a 1 nt Gap (5'-OH) collected at 1 s frame rate. Similar analysis was performed as described in (**a**), except the initial 635 nm laser exposure was used to photobleach Cy5 on the substrate (red arrow in Panel 1).

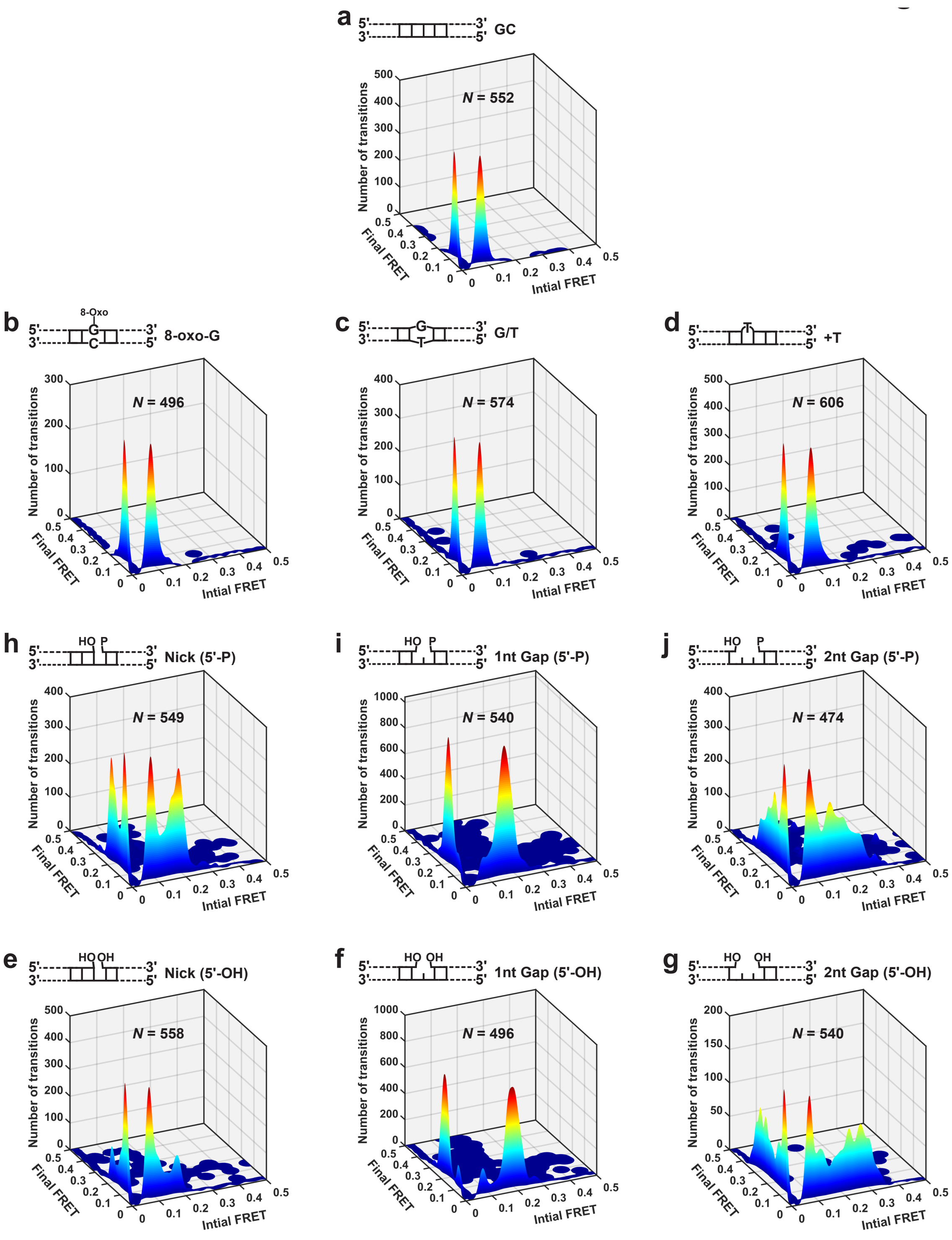

Supplementary Figure 3. Transition Density Plots for Cy3-PFV Target Capture Complex Formation with Different DNA Targets. (**a-g**) Transition density plots show the number of transitions between a given initial and a final FRET state. The identities of DNAs and the number of DNA molecules (*N*) used to build each transition density plots are shown.

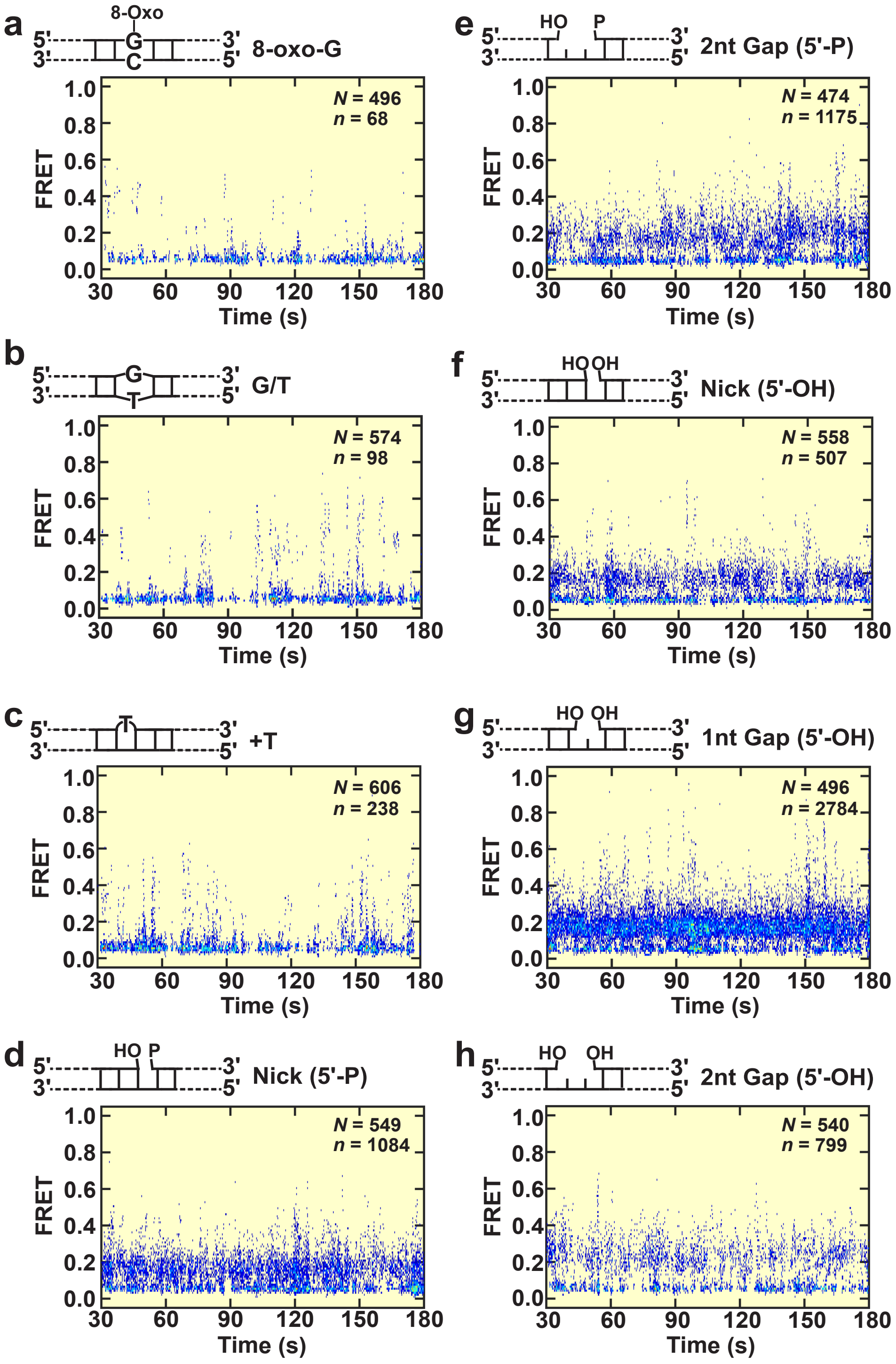

Supplementary Figure 4. Post-Synchronized Histograms of smFRET Trajectories Showing Cy3-PFV Target Capture Complex Formation on Different DNA Targets. (**a-h**) Post-synchronized histograms were generated by aligning smFRET trajectories from several (*N*) DNA molecules. The identities of DNAs and the total number of transitions (*n*) that crossed >0.1 FRET threshold is also shown.

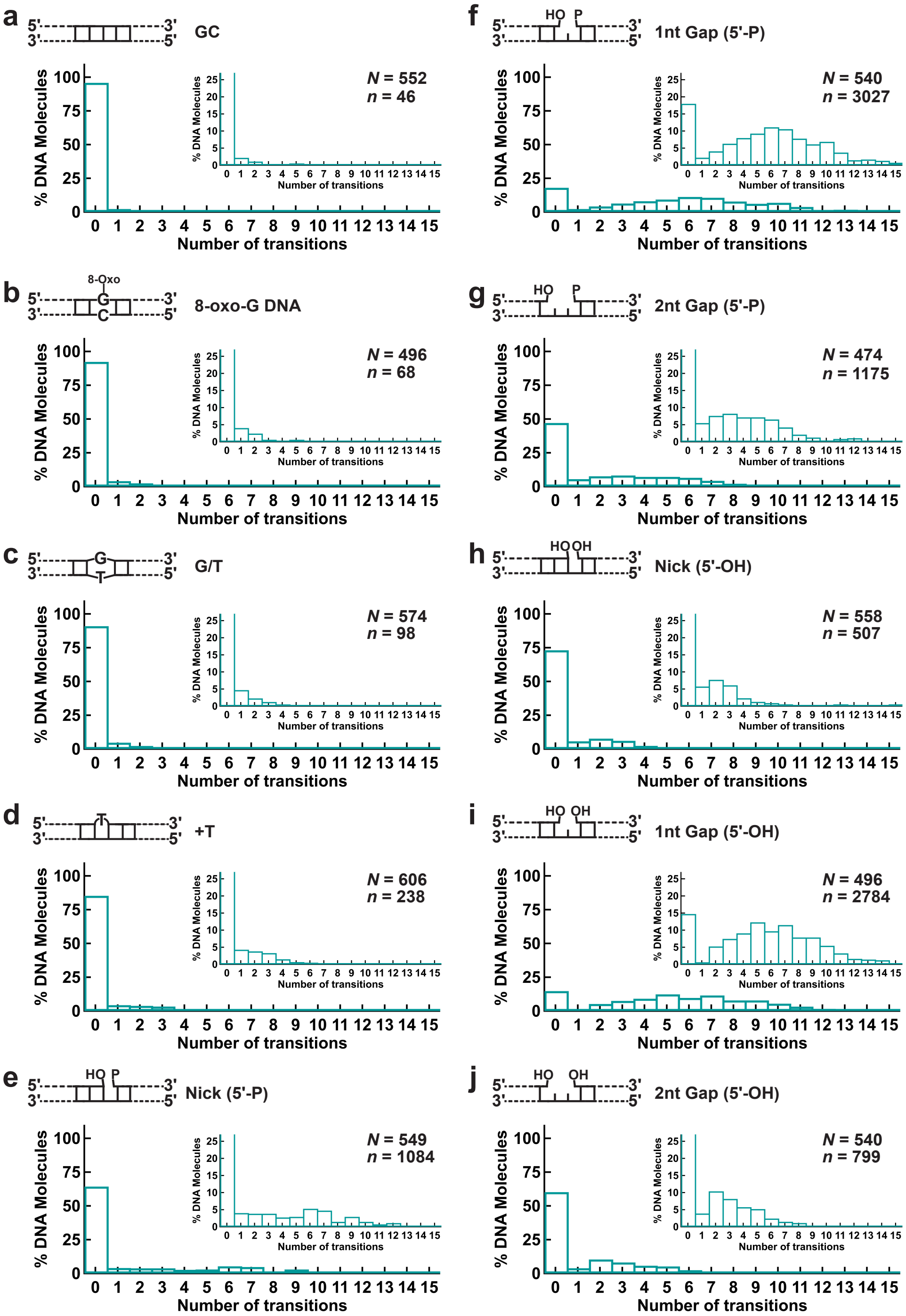

Supplementary Figure 5. Transition Count Histograms of Target Capture Complex Trajectories Collected at 100 ms Resolution. (**a-j**) Transition count histograms depict the fraction of DNA molecules that show a determined number of transitions during an observation window of 2.5 min. The identities of DNAs and the total number of DNA molecules (*N*) are shown. Inset is a 0-25% DNA molecules magnification for ease of substrate comparisons. Source data are provided in the Source Data File.

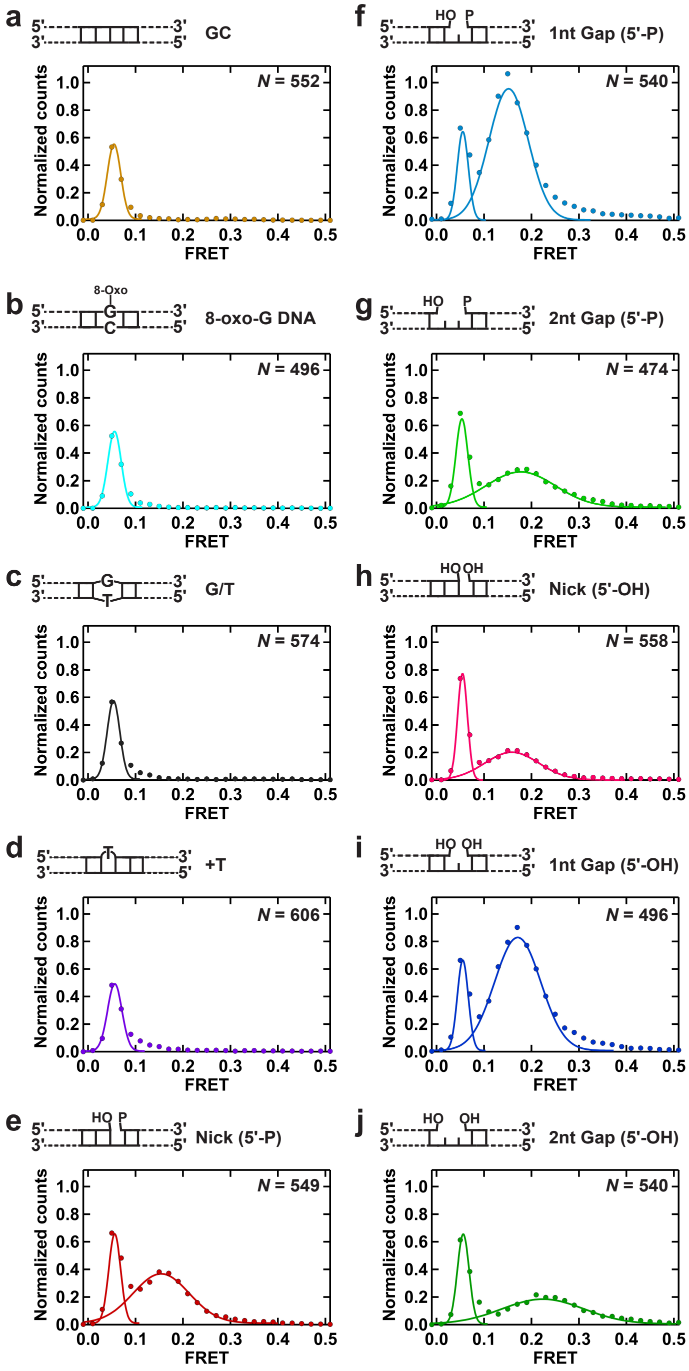

Supplementary Figure 6. Gaussian Fittings of the Target Capture Complex Histograms Collected at 100 ms Frame Rate. (**a-j**) The histograms were normalized with respect to the area under the pseudo (0.06) FRET peak as described in Online Methods. The normalized histograms for GC, 8-oxo-G, G/T, +T DNAs fit well with a single Gaussian. A combination of two Gaussians adequately models the histograms for the Nick, 1 nt and 2 nt Gap substrates, where the lower FRET peak corresponds to pseudo-FRET, and the higher FRET peak corresponds to intasome stalling on DNA near the lesion. The number of DNA molecules (*N*) is included in each histogram. Source data are provided in the Source Data File.

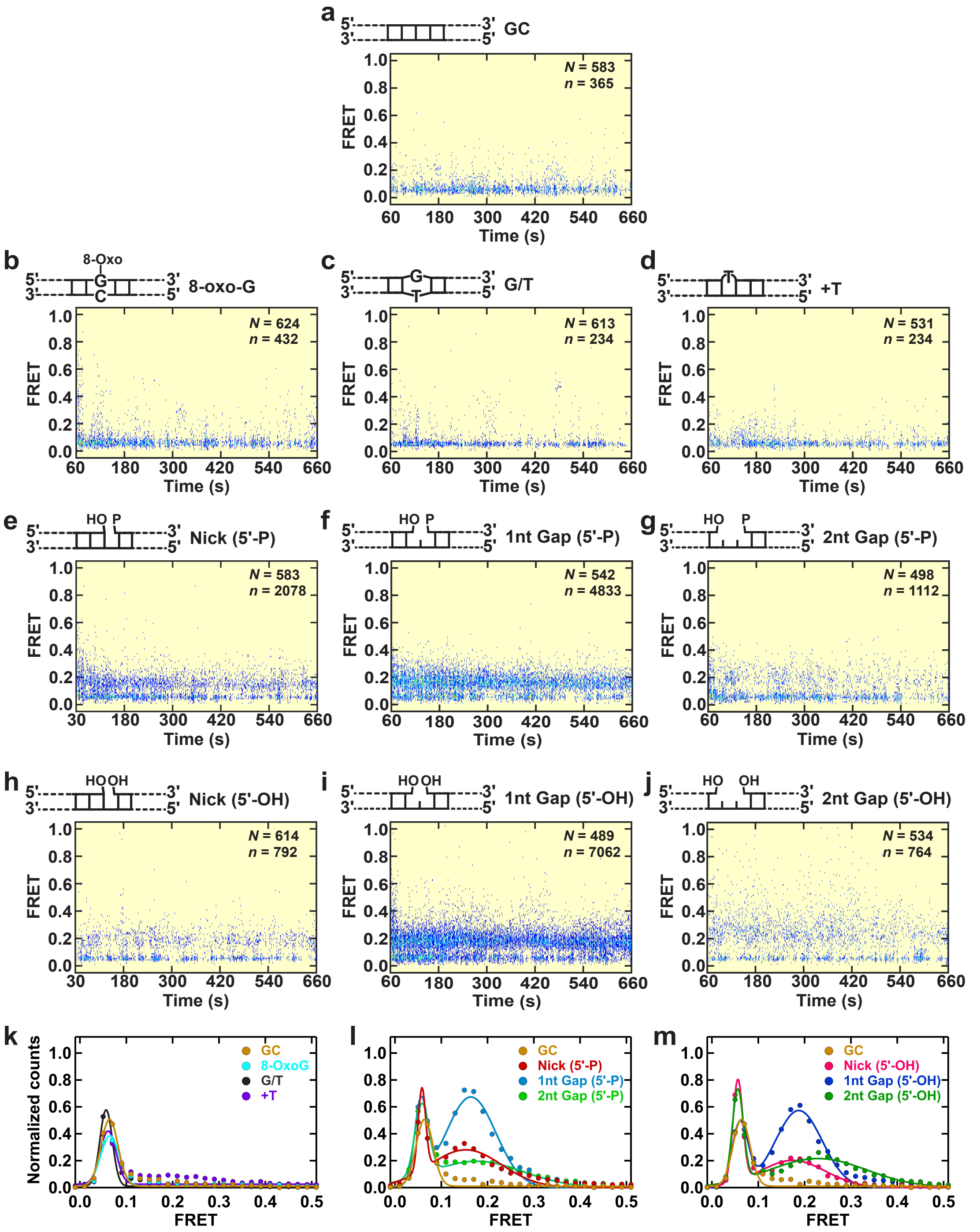

Supplementary Figure 7. PFV Intasome Target Capture Complex Dynamics at 1 s Frame Rate. (**a-j**) Post synchronized histograms displaying intasome interactions with different target DNA substrates. *N* is the number of DNA molecules analyzed for each substrate. *n* is the total number of transitions that crossed >0.1 FRET threshold. (**k**) Normalized smFRET histograms and their Gaussian fits showing the distributions of E_{pseudo} for GC, 8-oxo-G, G/T, +T DNAs. (**l,m**) Normalized smFRET histograms and their Gaussian fits showing the distributions of E_{pseudo} and E_{TCC} for (**l**) (5'-P) or (**m**) (5'-OH) Nick and Gap target DNA substrates. Source data are provided in the Source Data File.

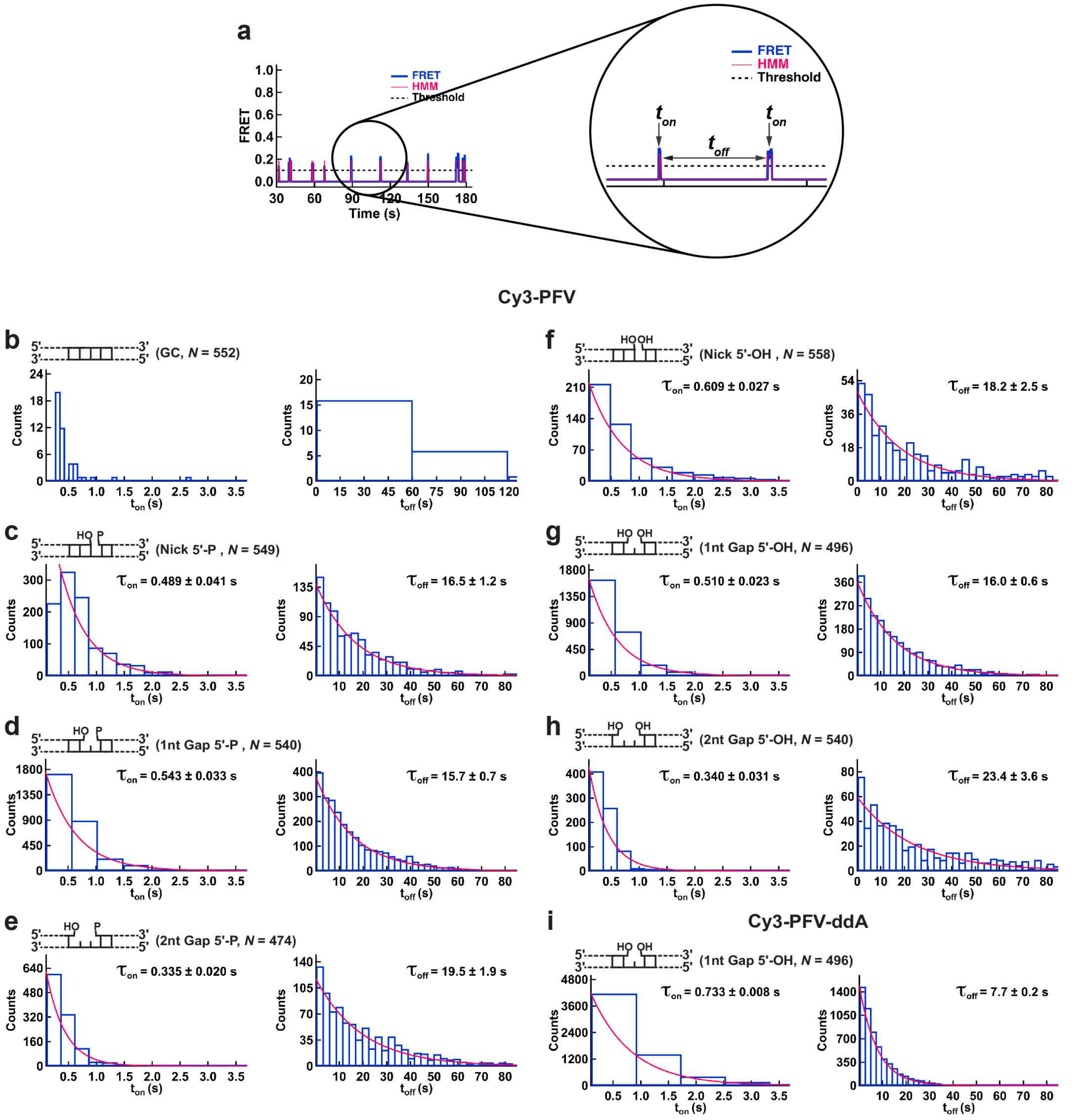

Supplementary Figure 8. Dwell-Time Analysis for PFV Intasome Target Capture Complex at 100 ms Frame Rate. (**a**) An illustration of FRET trajectories showing the transient TCC by Cy3-PFV intasomes. The magnified view illustrates the time spent in the bound (t_{on}) and the unbound (t_{off}) states. (**b-h**) Distributions of t_{on} and t_{off} for different target DNA substrates. (**i**) Distributions of t_{on} and t_{off} for Cy3-PFV-ddA intasomes on 1nt Gap (5'-OH) DNA. The single exponential fits (red lines), the resulting average dwell times, and the errors from the fittings are shown for each distribution. The identities of the DNAs and the number of molecules (*N*) used to build each histogram are also shown. Source data are provided in the Source Data File.

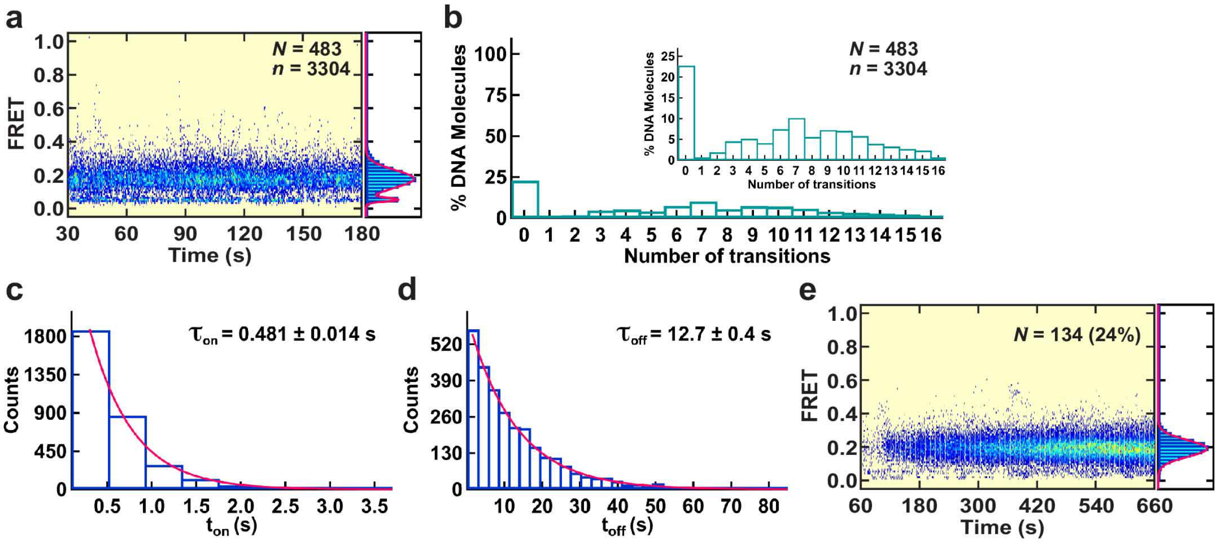

Supplementary Figure 9. Interactions of Cy3-PFV with Blocked End Target DNA. (**a**) The post synchronized histograms and the smFRET histogram corresponding to a collection of TCC events for blocked-end 1nt Gap (5'-OH) DNA. The Gaussian fits to the histograms is shown as a red line. (**b**) Transition count histogram showing the fraction of DNA molecules that showed a given number of transitions during the observation window of 3 min. (**c,d**) Distributions of t_{on} and t_{off} for the Target Capture Complex events. The single exponential fits (red lines), the resulting average dwell times, and the errors from the fittings are shown for each distribution. (**e**) The post synchronized histogram and the FRET histogram corresponding to *n* = 131 strand transfer events into blocked end 1nt Gap (5'-OH) DNA recorded at 1 sec frame rate. The frequency of strand transfer (%) is shown in parenthesis. Source data are provided in the Source Data File.

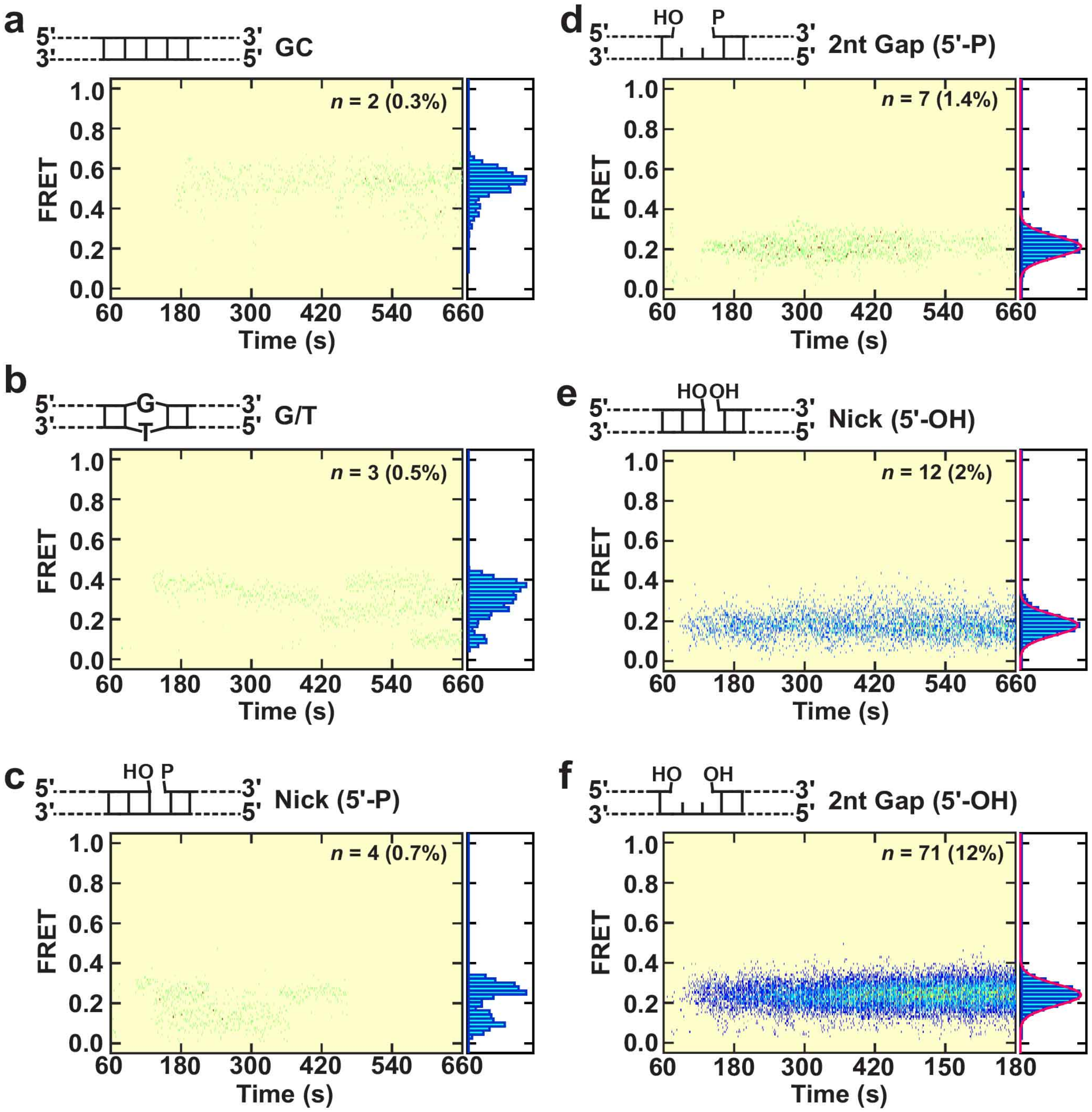

Supplementary Figure 10. Post Synchronized Histograms and smFRET Histograms of Cy3-PFV Strand Transfer Events. (**a-f**) The post synchronized histograms were generated by aligning smFRET trajectories that showed the formation of a strand transfer complex (STC). The identities of DNAs, the total number of strand transfer events (*n*) included in each analysis, and the efficiencies of STC formation (%) are shown. The Gaussian fits to the smFRET histograms are shown as red lines.

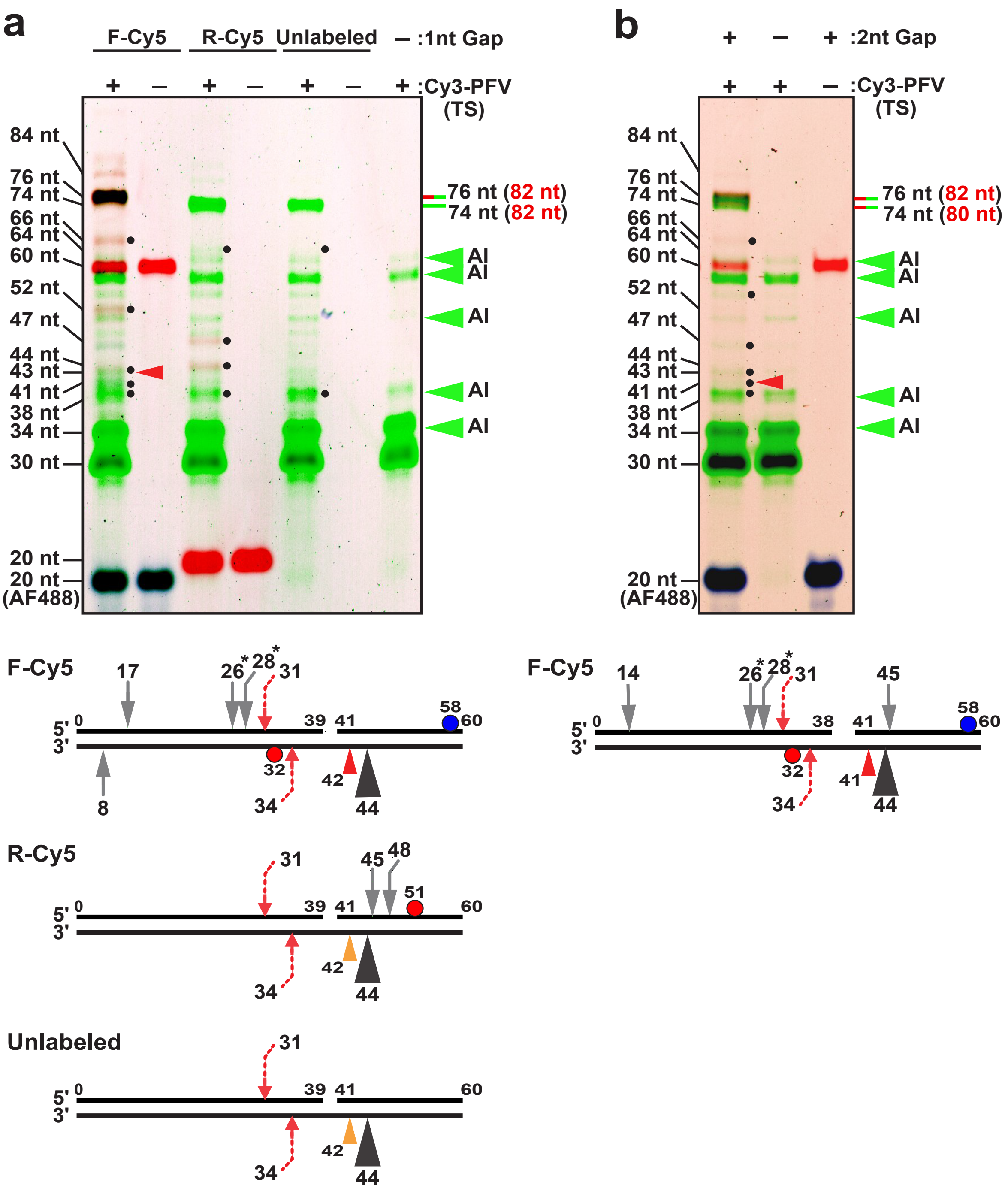

Supplementary Figure 11. Enhanced Image Contrast of Cy3-PFV (TS) Integration into 1 nt Gap (5'-OH) and 2 nt Gap (5'-OH) Target DNA. a) (top) Enhanced image contrast of 1 nt Gap (5'-OH) denaturing PAGE gel shown in Fig. 4d. Major half-site integration product of 74 nt and 76 nt (with Cy5 fluorophore that increases apparent size by 2 nt) are marked with green and green+red lines, which are equivalent to 82 nt integration product of unlabeled PFV intasome with the F-Cy5 target DNA shown in Fig. 4b,c. Dots next to gel band indicate integration products mapped to the DNA substrates illustrated below following subtraction of 30 nt corresponding to the ligated vDNA. Red arrowhead marks the 42 bp alcoholysis product (see Fig.

4b,c) on both the gel above and integration site illustration below (orange arrowhead in illustration shows the location of undetectable alcoholysis product since the corresponding DNA strand does not contain a fluorophore label). AI indicates auto-integration products found in both control and target DNA lanes. (bottom) Illustration F-Cy5, R-Cy5 and unlabeled target DNAs with calculated integration sites as arrowheads or arrows. The major half-site product maps to 44 nt on the non-lesion containing strand (black arrowhead), while the corresponding concerted integration event would occur at 40 nt on the lesion-containing strand where a phosphate bond required for the isoenergetic strand transfer is absent. Gray arrows in bottom illustration indicate the location of half-site products (* indicate most likely location, but also consistent with a second possible location at 11 and 13 nt on the same strand). Red dashed arrows show the location of the most frequent concerted integration product that appears with all three target DNAs. The 44 nt half-site event accounts for >90% of the products, while all other mapped events account for <5% of the total products. (**b**) Enhanced contrast of denaturing PAGE gel of Cy3-PFV integration products into F-Cy5 containing a 2 nt Gap (5'-OH). Markings of gel above and illustration below are similar to Panel a except with reference to 2 nt Gap shown in Fig. 4b,c. As with Panel a, the 44 bp half-site events accounts for >90% of the integration products.

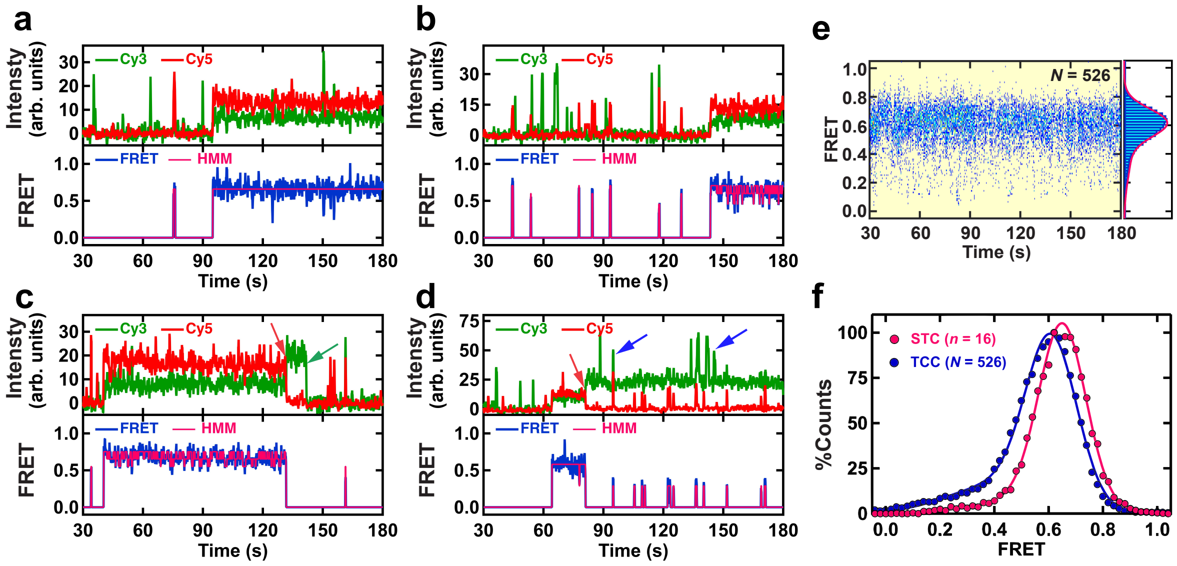

Supplementary Figure 12. Probing the Structural Dynamics of PFV Intasomes During Target Capture and Strand Transfer. (**a-d**) Representative intensity trajectories and the resultant FRET trajectories with the HMM fits showing strand transfer by Cy3/Cy5-PFV intasomes into 1nt Gap (5'-OH) DNA. For the longer STC FRET states, HMM analysis often fits fluctuations that are not originating from anti-correlated Cy3, Cy5 intensities. The photobleaching of Cy3 and Cy5 are denoted by green and red color arrows, respectively. The blue color arrows indicate aggregate excursions of the intasome that occasionally produce pseudo-FRET transitions. (**e**) Post-synchronized histogram (left) and smFRET histogram (right) of TCC FRET traces showing Cy3/Cy5-PFV binding to a 1nt Gap (5'-OH) target DNA. The data was collected at 100 msec frame rate. (**f**) smFRET histogram with the Gaussian fit for the STC (red) and TCC (blue) events exhibited by Cy3/Cy5-PFV intasome binding to 1nt Gap (5'-OH) DNA. Data was collected at 100 msec frame rate. The total number of DNA molecules (*N*) examined or the total number of STC events (*n*) are included in each histogram as indicated. Source data are provided in the Source Data File.
