# Supplementary material for: DNA strand breaks and gaps target retroviral intasome binding and integration
Source: Nat Commun. 2023 Nov 3;14:7072. doi: 10.1038/s41467-023-42641-4 (PMC10624929; doi:10.1038/s41467-023-42641-4)
Supplement: Supplementary file 3 — Description of Additional Supplementary Files [file 41467_2023_42641_MOESM3_ESM.pdf]

**Description of Additional Supplementary Files**

File Name: Supplementary Movie 1
Description: Representative movie showing single molecule FRET imaging of Cy3-PFV intasome interaction with a Cy5-duplex (G/C) DNA at 100 msec frame rate. Cy3 emission (left); Cy5 emission (right).

File Name: Supplementary Movie 2
Description: Representative movie showing single molecule FRET imaging of Cy3-PFV intasome interaction with a Cy5-1nt (5'-P) gap DNA at 100 msec frame rate. Cy3 emission (left); Cy5 emission (right).
