# Supplementary material for: DNA strand breaks and gaps target retroviral intasome binding and integration
Source: Nat Commun. 2023 Nov 3;14:7072. doi: 10.1038/s41467-023-42641-4 (PMC10624929; doi:10.1038/s41467-023-42641-4)
Supplement: Supplementary file 6 — Reporting Summary [file 41467_2023_42641_MOESM6_ESM.pdf]

nature portfolio

Corresponding author(s): Richard Fishel and Kristine E. Yoder

Last updated by author(s): Oct 9, 2023

Reporting Summary

Nature Portfolio wishes to improve the reproducibility of the work that we publish. This form provides structure for consistency and transparency in reporting. For further information on Nature Portfolio policies, see our Editorial Policies and the Editorial Policy Checklist.

Statistics

For all statistical analyses, confirm that the following items are present in the figure legend, table legend, main text, or Methods section.

n/a	Confirmed	
☐	☒	The exact sample size (*n*) for each experimental group/condition, given as a discrete number and unit of measurement
☐	☒	A statement on whether measurements were taken from distinct samples or whether the same sample was measured repeatedly
☒	☐	The statistical test(s) used AND whether they are one- or two-sided<br>*Only common tests should be described solely by name; describe more complex techniques in the Methods section.*
☒	☐	A description of all covariates tested
☒	☐	A description of any assumptions or corrections, such as tests of normality and adjustment for multiple comparisons
☐	☒	A full description of the statistical parameters including central tendency (e.g. means) or other basic estimates (e.g. regression coefficient) AND variation (e.g. standard deviation) or associated estimates of uncertainty (e.g. confidence intervals)
☒	☐	For null hypothesis testing, the test statistic (e.g. *F*, *t*, *r*) with confidence intervals, effect sizes, degrees of freedom and *P* value noted<br>*Give P values as exact values whenever suitable.*
☒	☐	For Bayesian analysis, information on the choice of priors and Markov chain Monte Carlo settings
☒	☐	For hierarchical and complex designs, identification of the appropriate level for tests and full reporting of outcomes
☒	☐	Estimates of effect sizes (e.g. Cohen's *d*, Pearson's *r*), indicating how they were calculated

Our web collection on statistics for biologists contains articles on many of the points above.

Software and code

Policy information about availability of computer code

Data collection	We utilized AzureSpot software (Azure Biosystems) for gel quantification; Single-molecule movies were acquired using the Micro-Manager imaging software.
Data analysis	Single-molecule imaging was analyzed with MATLAB R2019b (MathWorks) with custom code deposited in the database Zenodo under accession code 8411026 https://doi.org/10.5281/zenodo.8411026. This code was used to extract fluorophore traces, calculate FRET efficiency, perform vbFRET analysis, generate single-molecule trace plots, post-synchronized histograms, transition density plots, transition count histograms, and FRET efficiency histograms. The vbFRET software (Gonzalez Laboratory, Columbia University) was used to perform Hidden Markov Modeling.

For manuscripts utilizing custom algorithms or software that are central to the research but not yet described in published literature, software must be made available to editors and reviewers. We strongly encourage code deposition in a community repository (e.g. GitHub). See the Nature Portfolio guidelines for submitting code & software for further information.

Data

Policy information about availability of data

All manuscripts must include a data availability statement. This statement should provide the following information, where applicable:

- Accession codes, unique identifiers, or web links for publicly available datasets
- A description of any restrictions on data availability
- For clinical datasets or third party data, please ensure that the statement adheres to our policy

Two files from the database PDB under accession codes 3OS2 https://doi.org/10.2210/pdb3OS2/pdb and 3OS0 https://doi.org/10.2210/pdb3OS0/pdb were used to estimate FRET efficiencies.

Research involving human participants, their data, or biological material

Policy information about studies with human participants or human data. See also policy information about sex, gender (identity/presentation), and sexual orientation and race, ethnicity and racism.

Reporting on sex and gender	N/A
Reporting on race, ethnicity, or other socially relevant groupings	N/A
Population characteristics	N/A
Recruitment	N/A
Ethics oversight	N/A

Note that full information on the approval of the study protocol must also be provided in the manuscript.

Field-specific reporting

Please select the one below that is the best fit for your research. If you are not sure, read the appropriate sections before making your selection.

☒ Life sciences ☐ Behavioural & social sciences ☐ Ecological, evolutionary & environmental sciences

For a reference copy of the document with all sections, see nature.com/documents/nr-reporting-summary-flat.pdf

Life sciences study design

All studies must disclose on these points even when the disclosure is negative.

Sample size	No statistical method was used to predetermine sample size. For single-molecule imaging we collected as many data points as possible with in our camera's field of view which we found to be sufficient to produce reproducible results. For gels we replicated each gel at least twice.
Data exclusions	No data was excluded from the analysis.
Replication	All experiments were performed at least twice, on separate days, with separate batches of reagents. All replications were successful.
Randomization	No experimental groups were used in this study.
Blinding	Blinding was not relevant since there were no experimental group used in this study.

Reporting for specific materials, systems and methods

We require information from authors about some types of materials, experimental systems and methods used in many studies. Here, indicate whether each material, system or method listed is relevant to your study. If you are not sure if a list item applies to your research, read the appropriate section before selecting a response.

Materials & experimental systems

n/a	Involved in the study	
☒	☐	Antibodies
☒	☐	Eukaryotic cell lines
☒	☐	Palaeontology and archaeology
☒	☐	Animals and other organisms
☒	☐	Clinical data
☒	☐	Dual use research of concern
☒	☐	Plants

Methods

n/a	Involved in the study	
☒	☐	ChIP-seq
☒	☐	Flow cytometry
☒	☐	MRI-based neuroimaging
